# Identification of BALB/c Immune Markers Correlated with a Partial Protection to *Leishmania infantum* after Vaccination with a Rationally Designed Multi-epitope Cysteine Protease A Peptide-Based Nanovaccine

**DOI:** 10.1371/journal.pntd.0005311

**Published:** 2017-01-23

**Authors:** Maria Agallou, Maritsa Margaroni, Evita Athanasiou, Dimitra K. Toubanaki, Katerina Kontonikola, Konstantina Karidi, Olga Kammona, Costas Kiparissides, Evdokia Karagouni

**Affiliations:** 1 Department of Microbiology, Hellenic Pasteur Institute, Athens, Greece; 2 Chemical Process & Energy Resources Institute, Centre for Research and Technology Hellas, Thessaloniki, Greece; 3 Department of Chemical Engineering, Aristotle University of Thessaloniki, Thessaloniki, Greece; Queensland Institute of Medical Research, AUSTRALIA

## Abstract

**Background:**

Through their increased potential to be engaged and processed by dendritic cells (DCs), nanovaccines consisting of Poly(D,L-lactic-co-glycolic acid) (PLGA) nanoparticles (NPs) loaded with both antigenic moieties and adjuvants are attractive candidates for triggering specific defense mechanisms against intracellular pathogens. The aim of the present study was to evaluate the immunogenicity and prophylactic potential of a rationally designed multi-epitope peptide of *Leishmania* Cysteine Protease A (CPA_160-189_) co-encapsulated with Monophosphoryl lipid A (MPLA) in PLGA NPs against *L*. *infantum* in BALB/c mice and identify immune markers correlated with protective responses.

**Methodology/Principal Findings:**

The DCs phenotypic and functional features exposed to soluble (CPA_160-189_, CPA_160-189_+MPLA) or encapsulated in PLGA NPs forms of peptide and adjuvant (PLGA-MPLA, PLGA-CPA_160-189_, PLGA-CPA_160-189_+MPLA) was firstly determined using BALB/c bone marrow-derived DCs. The most potent signatures of DCs maturation were obtained with the PLGA-CPA_160-189_+MPLA NPs. Subcutaneous administration of PLGA-CPA_160-189_+MPLA NPs in BALB/c mice induced specific anti-CPA_160-189_ cellular and humoral immune responses characterized by T cells producing high amounts of IL-2, IFN-γ and TNFα and IgG1/IgG2a antibodies. When these mice were challenged with 2x10^7^ stationary phase *L*. *infantum* promastigotes, they displayed significant reduced hepatic (48%) and splenic (90%) parasite load at 1 month post-challenge. This protective phenotype was accompanied by a strong spleen lymphoproliferative response and high levels of IL-2, IFN-γ and TNFα versus low IL-4 and IL-10 secretion. Although, at 4 months post-challenge, the reduced parasite load was preserved in the liver (61%), an increase was detected in the spleen (30%), indicating a partial vaccine-induced protection.

**Conclusions/Significance:**

This study provide a basis for the development of peptide-based nanovaccines against leishmaniasis, since it reveals that vaccination with well-defined *Leishmania* MHC-restricted epitopes extracted from various immunogenic proteins co-encapsulated with the proper adjuvant or/and phlebotomine fly saliva multi-epitope peptides into clinically compatible PLGA NPs could be a promising approach for the induction of a strong and sustainable protective immunity.

## Author Summary

Leishmaniases are a wide spectrum of parasite diseases caused by different species of the genus *Leishmania*. Among them, visceral leishmaniasis is the most severe form of the disease affecting millions of people worldwide. Currently chemotherapeutic agents are highly toxic and suffer from extensive drug resistance. However, the development of a safe and effective vaccine has to deal with the complications erased from the different immune responses of the human population. It is suggested that a solution to that problem is the design of candidate vaccines containing appropriate multi-epitope peptides for the induction of protective immunity. Furthermore, the selection of proper adjuvant and biocompatible delivery systems for the peptide(s) chosen should enhance their immunogenic potential. Subsequently, for this work, we used a synthetic peptide designed to contain overlapping epitopes obtained from the sequence of a known immunogenic *Leishmania* protein, Cysteine Protease A (CPA). The peptide was selected to be delivered along with MPLA adjuvant co-encapsulated in PLGA nanoparticles. The data presented in this study show the immunogenicity and the prophylactic potential of the proposed nanovaccine against *L*. *infantum* in the susceptible model of visceral leishmaniasis in BALB/c mice, further suggesting that rationally designed peptide-based nanovaccines are promising vaccine candidates against leishmaniasis.

## Introduction

Leishmaniasis is an infectious diseases complex caused by protozoan parasites of the genus *Leishmania*. According to epidemiological data, visceral leishmaniasis (VL) is the second most common parasitic cause of death among tropical infections and is prevalent in 47 countries with about 200 million people at risk and an estimated annual incidence of approximately 500,000 cases [1,2]. Despite the fact that vaccination is considered to be the most promising and effective strategy for controlling leishmaniasis, to date no efficacious vaccine exists against human VL. First attempts at developing an anti-leishmanial vaccine were based on the injection of live virulent *L*. *major* parasites in healthy people, a process known as "leishmanization”. However, this process was discontinued due to safety and ethical reasons and replaced by first-generation vaccines composed by attenuated or inactivated pathogens or even pathogen subunits that in many cases showed inconsistent clinical outcomes [3,4,5].

Subsequently, many research efforts are focused on the development of second generation vaccines that are consisted of recombinant proteins or defined peptides. To date many different *Leishmania* antigens have been found to be potential vaccine candidates delivered by a plethora of immunizations regimens in animal models. However, these promising findings were overshadowed by mostly negative T-cell responses in humans [6,7]. During the last few years, remarkable advancements in immunoinformatics science have improved the selection of potential immunogenic epitopes from various pathogens. This coupled with *in vitro* immunogenicity testing of predicted peptides using exposed human blood samples may accelerate the development of candidate vaccines for leishmaniasis [7,8,9,10,11]. However, a major limiting factor for these poly-epitope peptide-based vaccines is their relatively low immunogenicity and their inability to trigger long-term immunity.

Previous studies proposed the encapsulation of whole proteins, soluble antigen or parasites in different nanoformulations in order to achieve a sustained antigen release for the development of strong and long-lasting T cell responses against leishmaniasis [12,13]. Among developed nanoparticles (NPs) Poly(D,L-lactic-co-glycolide) (PLGA) NPs are considered potent candidates for vaccine delivery systems due to their excellent safety profile, high encapsulation efficiency, tissue bio-distribution, controlled release pattern and their effectiveness to induce appropriate immune responses [14,15,16,17]. Moreover, the immunomodulatory properties of these particles can be significantly enhanced through the addition of adjuvants, such as Toll-like receptor (TLR) ligands [18]. A most common adjuvant used is Monophosphoryl lipid A (MPLA), a non-toxic derivative of the lipopolysaccharide (LPS) of *Salmonella minnesota*. MPLA is a well-tolerated adjuvant approved for human use which signals through TLR4 for the activation of T cell effector responses [19].

A vaccine against leishmaniasis is considered effective when it ensures a long-lasting cell-mediated immunity [20]. In VL, protective vaccination requires the activation of the innate arms of host defense consisting of macrophages and DCs resulting in a long-term activation of both CD4^+^ helper and CD8^+^ effector T cells [21]. For that reason, research efforts on vaccine development are focused on the identification of recombinant proteins or defined peptides, capable to induce appropriate immune responses [6,7]. Among them, Cysteine Protease A (CPA) is a conserved protein expressed not only in the sand fly promastigote stage, but more importantly, in the disease-causing mammalian amastigotes [22,23]. Furthermore, it has been shown to play an important role in the immunity against *Leishmania*, since it is recognized by sera obtained from either recovered or active cases of CL and VL, as well as by sera from asymptomatic or symptomatic dogs with leishmaniasis [24,25,26]. In a recent study, we designed a 30-mer multi-epitope peptide (CPA_160-189_) that contained 3 and 2 overlapping MHC class I and II-restricted epitopes, respectively, obtained from *L*. *infantum* CPA sequence by using *in silico* analysis. Furthermore, we showed that immunization of *Leishmania*-susceptible BALB/c mice with CPA_160-189_ in emulsion with Freund’s adjuvant elicited peptide-specific CD4^+^ Th1 and CD8^+^ effector T cell immune responses that are required for protection against VL [27].

The aim of the present study was to improve peptide’s immunogenicity by co-encapsulating it with MPLA adjuvant into PLGA NPs and evaluate its prophylactic potential against VL. For this purpose, analysis of the potentiating effects of this formulation in phenotypic and functional features of BALB/c bone marrow-derived DCs and its ability to induce strong T cell immunity against *L*. *infantum* was determined. Evidence presented from both *in vitro* and *in vivo* settings suggests that the development of a peptide-based nanovaccine consisting of a rationally designed multi-epitope peptide and a suitable adjuvant could be a promising tool to prevent VL.

## Methods

### Ethics statement

Animal experiments were performed in strict accordance with the National Law 2013/56, which adheres to the European Directive 2010/63/EU for animal experiments and complied with the ARRIVE guidelines. The protocol was approved by the institutional Animal Bioethics Committee (Approval Number: 4455/10-07-2014). All efforts were made to minimize animal suffering. Serum samples from domestic dogs (*Canis familiaris*) were obtained from an already-existing biobank of our laboratory. Also, *L*. *infantum* used in the present study was obtained from the already-existing “*Leishmania* cryobank collection” of the Hellenic Pasteur Institute. All samples were coded and anonymized. No IRB approval was required for using the strain.

### Animals, parasites and preparation of soluble antigen

Studies were performed with female 6–8 weeks old BALB/c mice reared in the pathogen-free animal care facility at Hellenic Pasteur Institute. They were housed in a climatically controlled room receiving a diet of commercial food pellets and water *ad libitum*.

A strain of *L*. *infantum* (MHOM/GR/2001/GH8) originally isolated from a Greek patient suffering from VL [28] was cultured *in vitro* and was maintained infective through serial passage in BALB/c mice, as described elsewhere [29]. The promastigote form of the parasite was cultured at 26°C in RPMI-1640 medium (Biochrom AG, Berlin, Germany) supplemented with 2 mM L-glutamine, 10 mM HEPES, 24 mM NaHCO_3_, 100 U/ml penicillin, 10 μg/ml streptomycin and 10% (v/v) heat inactivated fetal bovine serum (FBS; Gibco, Paisley, UK).

For preparation of soluble *Leishmania* antigen (SLA), 1x10^9^ late-log phase promastigotes were washed thrice in PBS and disrupted by five repeated freezing/thawing cycles (liquid N_2_/37°C) followed by 5 min incubation on ice. Partially lysed material was exposed for 30 sec in a sonicator (UP100H, Hielscher Ultrasonics GmbH, Teltow, Germany) and then centrifuged for 30 min at 8,000×g at 4°C. The supernatant containing SLA was collected and total protein concentration was determined spectrophotometrically using the MicroBCA Protein Assay Kit (Thermo Scientific, Rockford, IL, USA) at 570 nm. SLA was stored at -80°C in aliquots until use.

### Canine serum samples and detection of anti-CPA_160-189_ antibodies

For the evaluation of CPA_160-189_ immunogenicity, peptide-specific IgG antibodies were detected in serum samples of naturally infected with *L*. *infantum* asymptomatic (n = 12) or symptomatic (n = 14) and healthy dogs (n = 6) [30]. For the detection of CPA_160-189_- or parasite-specific IgG antibodies, 96-well microtiter plates were coated with 5 μg/ml CPA_160-189_ or SLA, respectively, and incubated overnight at 4°C. After 3 washes with washing buffer (PBS with 0.05% Tween 20), plates were blocked with blocking buffer (2% BSA in PBS) for 2 h at 37°C and then 100 μl of dog sera were added at 1:400 dilution, and incubated for another 2 h at 37°C. HRP-conjugated anti-dog IgG were added and incubated for 1 h at 37°C. The enzyme-labeled complexes were detected by reaction with TMB substrate (Thermo Scientific) and the reaction was stopped by adding 2 M sulfuric acid. The absorbance was measured at 450 nm using an ELISA microplate spectrophotometer (MRX, Dynatech Laboratories, Guernsey, UK).

### Preparation of PLGA NPs

For NPs synthesis, PLGA 75:25 (Resomer RG752H, MW: 4–15 kDa), polyvinyl alcohol (PVA) (MW: 30–70 kDa, 87–90% hydrolyzed) and MPLA from *Salmonella minnesota* were purchased from Sigma-Aldrich (Vienna, Austria). CPΑ_160–189_ peptide obtained from the sequence of *L*. *infantum* CPA protein (GenBank Acc. No.: CAM67356) was synthesized by GeneCust (Labbx, Dudenange, Luxenmbourg) with purity ≥95%. All other reagents were of analytical grade and commercially available. PLGA NPs containing the peptide CPΑ_160–189_ and the adjuvant MPLA were prepared by the double emulsion method, as described previously [31]. Briefly, 2.9 ml of a PLGA chloroform solution (31 mg/ml) were mixed with 0.1 ml of an MPLA solution (10 mg/ml) in methanol:chloroform (1:4 v/v). A water-in-oil (w/o) emulsion was then formed by adding 0.3 ml of the peptide solution in PBS at a final concentration of 6.6 mg/ml into the PLGA/MPLA solution. The emulsification was performed in an ice bath with the aid of a microtip sonicator (Sonicator Sonics Vibra Cell VC-505, Sonics, Newtown, CT, USA) at 40% amplitude for 45 sec. Subsequently, the primary emulsion (w/o) was added into 12 ml of 1% (w/v) aqueous PVA solution. The mixture was then emulsified via sonication at 40% amplitude for 2 min. The resulting double (w/o/w) emulsion was stirred overnight to allow the evaporation of chloroform. The PLGA NPs were then purified by means of four successive centrifugation-redispersion cycles in sterilized water, at 13,860×g for 10 min at 4°C and were subsequently lyophilized (ScanVac Freezedryers CoolSafe 55–9, Scientific Laboratory Supplies, Yorkshire, UK). For the preparation of the PLGA-CPΑ_160–189_ NPs, the same volume of peptide solution as previously was added into 3 ml of a PLGA/chloroform solution at a final concentration of 30 mg/ml. Finally, to prepare blank PLGA NPs for controls, the CPΑ_160–189_ aqueous solution was replaced with 0.3 ml of PBS. Lyophilized PLGA NPs were stored at 4°C.

The surface morphology of the synthesized PLGA NPs was observed by scanning electron microscopy (SEM) (JEOL JSM 6300, Jeol, Peabody, MA, USA). Accordingly, the lyophilized NPs were first double coated with a gold layer under vacuum and then examined by SEM. The average particle diameter of the PLGA NPs was determined by photon correlation spectroscopy and their zeta potential by aqueous electrophoresis measurements (Malvern Nano ZS90, UK). The measurements were performed with aqueous dispersions of NPs prior to lyophilization.

### Determination of peptide and adjuvant loadings

The MicroBCA Protein assay kit (Thermo Scientific) was employed to determine CPΑ_160–189_ encapsulation (μg/mg) in the PLGA NPs. Accordingly, 2.5 mg of lyophilized PLGA NPs were dissolved in 0.25 ml DMSO for 1 h following a further dissolution in 1.25 ml of 0.05 M NaOH/0.5% SDS for 3 h at 25°C. Blank PLGA NPs were used as negative controls. The absorbance of the samples was measured at 562 nm using a microplate reader (EL808IU-PC, BioTek Instruments, Inc., Winooski VT, USA). Antigen encapsulation efficiency (EE) was calculated by the ratio of the antigen mass in the NPs over the total mass of antigen used. Also, the antigen loading was calculated by the ratio of the encapsulated mass of antigen over the total mass of PLGA NPs.

A Limulus Amebocyte Lysate (LAL) kit (Thermo Scientific) was used for the determination of the MPLA loading (μg/mg) in the PLGA NPs. Standard curve was established using different concentrations of aqueous MPLA solutions ranging from 0.01 to 10 ng/ml, which was found to be linear for the MPLA concentration range used, with a correlation coefficient of R^2^ = 0.9994. The encapsulation efficiency of MPLA was calculated by the ratio of the measured MPLA mass in the NPs over the total mass of MPLA used and the MPLA loading was calculated by the ratio of encapsulated MPLA mass over the total mass of the PLGA NPs.

For the determination of *in vitro* release of CPΑ_160–189_ and MPLA, PLGA NPs were dispersed in PBS at a final concentration of 1 mg/ml and were incubated at 37°C under constant shacking at 120.7×g. At predetermined time points (0, 1, 2, 4, 6, 8, 12, 24, 48 h, 1 and 2 weeks) 1 ml of the dispersion was centrifuged at 13,860×g for 10 min at 4°C. Then, the supernatants were collected and the amount of CPΑ_160–189_ and MPLA were determined using the MicroBCA and LAL kits, respectively.

### Generation of bone marrow-derived DCs

DCs were generated from pluripotent bone marrow stem cells of BALB/c mice in the presence of rmGM-CSF, as reported previously [29]. On day 7, non-adherent and semi-adherent cells were collected and phenotypic analysis was performed by flow cytometry using antibodies against CD11c and CD8a surface markers. According to trypan blue exclusion, cell viability was >95% and the percentage of CD11c^+^CD8a^-^ cells was >75%, as assayed by FACS analysis.

### Flow cytometry analysis for the detection of DCs maturation and cytokine production

DCs maturation induced by PLGA NPs was determined by flow cytometry. All antibodies used were obtained from BD Biosciences (Erembodegem, Belgium). For this purpose, DCs were cultured into a 24-well plate at a density of 1x10^6^ cells/ml/well in the presence of PLGA-MPLA, PLGA-CPA_160-189_ or PLGA-CPA_160-189_+MPLA NPs, or soluble CPΑ_160–189_ in the presence or not of soluble MPLA at various doses for 24 h at 37°C in a humidified CO_2_ incubator. DCs in medium alone and DCs stimulated with LPS (1 μg/ml) were used as negative and positive control, respectively. At the end of incubation, wells were washed to remove free PLGA NPs followed by a wash with FACS buffer (PBS– 3% (v/v) FBS). The cells were then labeled with PE-conjugated anti-mouse CD40, CD80, CD83, CD86, MHCI (1:100 dilution) or MHCII (1:200 dilution) mAbs for 30 min at 4°C. For intracellular staining, cells were subjected to brefeldin A (2.5 μg/ml) for the last 4 h of culture and then were fixed with 2% paraformaldehyde and stained with PE-conjugated IL-12p40 mAb (1:100 dilution) in permeabilization buffer (FACS buffer supplemented with 0.1% saponin). After staining, cells were washed with FACS buffer and subjected to flow cytometric analysis using a FACS Calibur system (Becton-Dickinson, San Jose, CA, USA). Data were analyzed using FlowJo software version 10.0 (Tree Star, Inc., Ashland, OR, USA).

### *In vitro* T cell priming assay

DCs (1x10^6^ cells/ml) that had been pulsed with medium, PLGA-CPA_160-189_ or PLGA-CPA_160-189_+MPLA NPs for 24 h were co-cultured with naive splenocytes of similar origin, at a responder/stimulator ratio of 5:1 in a 96-well round-bottom plate for 96 h. Splenocytes cultured in medium alone or in the presence of Con A (6 μg/ml; Sigma) served as negative or positive control of T cell proliferation, respectively. Proliferation was determined by addition of 0.5 μCi of [^3^H]-thymidine ([^3^H]-TdR; PerkinElmer, Boston, MA, USA) during the last 18 h of the culture period and subsequent measurement of [^3^H]-TdR incorporation on a microplate scintillation and luminescence counter (Microbeta Trilux, Wallac, Turcu, Finland). All assays were performed in triplicates. Stimulation index (S.I.) was calculated according to the following formula: S.I. = cpm measured in T cells in the presence of pulsed DCs / cpm measured in T cells cultured in medium alone (negative control).

### Real time quantitative PCR for the detection of cytokine expression

IFN-γ, IL-4 and IL-10 mRNA expression by DCs-stimulated T cells was determined by real time quantitative PCR (qPCR). Specifically, complementary DNA (cDNA) was synthesized using RT^2^ HT First Strand Kit (Qiagen, Maryland, USA) from 1 μg of total RNA isolated from cells from each culture using the RNeasy mini kit (Qiagen) in GeneAmp PCR System 9700 (Applied Biosystems, NY, USA). Determination of IFN-γ, IL-4 and IL-10 mRNA levels was carried out by a SYBR green real time qPCR using a custom RT^2^ Profiler PCR Array (CAPM13028, Qiagen) in a Stratagene Mx3005P PCR System (Agilent Technologies, Santa Clara, CA, USA), according to manufacturer’s instructions. Mouse Glyceraldehyde-3-phosphate dehydrogenase (GAPDH) was used as an endogenous control to avoid variations between the samples. Briefly, cDNA samples were subjected to an initial denaturation for 10 min at 95°C, followed by 40 cycles of denaturation at 95°C for 15 sec, and extension at 60°C for 30 sec, ending with incubation at 72°C for 5 min. Each reaction was carried out in triplicate. Non-pulsed DCs-stimulated T cells were used as calibrators and results were expressed as fold change by the ΔΔCt method. Briefly, the normalized to GAPDH gene expression (2^-ΔΔCt^) in cultures of interest were divided by the normalized to GAPDH gene expression (2^-ΔΔCt^) in the calibrators, where value of 1 is the gene expression baseline in non-pulsed DCs-stimulated T cell cultures. A non-template control without genetic material was included to eliminate nonspecific reactions.

## Vaccination schedules with peptide-based nanovaccines and challenge infection

Groups of BALB/c mice (n = 25/group) were vaccinated subcutaneously in the upper and dorsal region with (i) PLGA-MPLA NPs, (ii) PLGA-CPA_160-189_ NPs or (iii) PLGA-CPA_160-189_+MPLA NPs in a total volume of 100 μl sterile PBS, as described in Table 1. Taken into account the peptide and MPLA loadings, each mouse received 2 μg of peptide with or without 1 μg MPLA or 1 μg MPLA alone. Two booster doses followed at a 2 weeks interval. Mice receiving PBS alone served as control. Two weeks after each injection, mice (n = 5/group) were euthanized and sera samples and spleens were collected to analyze the immune response induced by vaccination. Two weeks after the final boost, the remaining vaccinated and non-vaccinated mice were challenged by injecting freshly transformed 2x10^7^ stationary phase *L*. *infantum* promastigotes intravenously. Non-vaccinated non-infected mice served as negative control. The prophylactic efficacy of PLGA NPs formulations was assessed in spleen and liver at 1 and 4 months post challenge. The percentage of inhibition of parasite multiplication was calculated in comparison with the non-vaccinated control using the following formula: percentage of inhibition = (number of parasites from non-vaccinated infected control group–number of parasites from the vaccinated infected group / number of parasites from non-vaccinated infected control group) x 100.

**Table 1:** Vaccination and challenge schedule in BALB/c mice.

Day^a^	Group 1	Group 2	Group 3	Group 4 (Non-vaccinated infected mice)^e^	Group 5 (Negative control)^e^
0	PLGA-CPA_160-189_ NPs^b^	PLGA-CPA_160-189_+MPLA NPs^c^	PLGA-MPLA NPs^d^	-	-
14	PLGA-CPA_160-189_ NPs^b^	PLGA-CPA_160-189_+MPLA NPs^c^	PLGA-MPLA NPs^d^	-	-
28	PLGA-CPA_160-189_ NPs^b^	PLGA-CPA_160-189_+MPLA NPs^c^	PLGA-MPLA NPs^d^	-	-
42	2x10^7^ *L*. *infantum*/mouse	2x10^7^ *L*. *infantum*/mouse	2x10^7^ *L*. *infantum*/mouse	2x10^7^ *L*. *infantum*/mouse	-

^a^Subcutaneous route for vaccination and intravenous route for parasite challenge.

^b^2 μg of encapsulated CPA_160-189_ were given per dose.

^c^2 μg of encapsulated CPA_160-189_ and 1 μg of encapsulated MPLA were given per dose.

^d^1 μg of encapsulated MPLA was given per dose.

^e^Mice received subcutaneously 100 μl of sterile PBS.

### Evaluation of peptide-based nanovaccines toxicity and induction of inflammation in mice

In order to examine *in vivo* toxicity of the synthesized PLGA NPs, BALB/c mice (n = 5/group) were injected subcutaneously at the indicated doses in 100 μl PBS. BALB/c mice (n = 5/group) injected with PBS or LPS (1 μg) served as negative or positive control of inflammation, respectively. The viability and the behavior of mice were observed at predetermined intervals over a 3 days post injection period. Furthermore, for the determination of inflammatory mediators, blood was collected from mice (n = 5/group) 4 h post vaccination. Sera were analyzed for IL-1β, IL-6, TNFα and MCP-1 by magnetic bead multiplex array (Millipore, Billerica, MA, USA). Data were acquired on a Luminex 200 (Oosterhoot, The Netherlands) and analyzed using xPONENT software (Luminex).

### Evaluation of parasite load by limiting dilution assay

The parasite burden was quantified by a limiting dilution assay as described previously [32]. The wells were examined for viable and motile promastigotes every 3 days, and the reciprocal of the highest dilution that was positive for parasites was considered to be the number of parasites per mg of tissue. The total parasite burden was calculated by reference to the whole organ weight.

### Spleen cell lymphoproliferation and determination of cytokine production

Spleens were aseptically excised from mice of all experimental groups two weeks after each vaccination (Table 1) as well as 1 month post challenge, and single cell suspensions were prepared in complete RPMI-1640. Cells were cultured in triplicates in 96-well round bottom plate at a density of 2x10^5^ cells/well at a final volume of 200 μl and stimulated with CPA_160-189_ (5 μg/ml) or SLA (12.5 μg/ml) for 96 h. Splenocytes cultured in medium alone or in the presence of Con A (6 μg/ml; Sigma-Aldrich, St. Louis, MO, USA) served as negative or positive control, respectively. Proliferation was determined by addition of 0.5 μCi of [^3^H]-thymidine ([^3^H]-TdR; PerkinElmer) during the last 18 h of the culture period.

In parallel, similar spleen cell cultures were performed for cytokine production 2 weeks after the end of vaccinations and 1 month post challenge. Specifically, spleen cells (1x10^6^ cells/ml) from all groups of mice were plated in 24-well plates and incubated with CPA_160-189_ (5 μg/ml) or SLA (12.5 μg/ml) for 72 hours. At the end of incubation period, cell-free supernatants were harvested by centrifugation, aliquoted and stored at -70°C until assayed for specific cytokines. The levels of IL-2, IFN-γ, TNFα, IL-4, and IL-10 were measured by magnetic bead multiplex array (Millipore).

### Flow cytometry analysis for determination of IFN-γ-producing cells

For intracellular analysis of IFN-γ-producing CD4^+^ and CD8^+^ T lymphocytes from all groups of mice at the end of vaccinations, flow cytometry analysis was carried out. In brief, two weeks after the end of vaccinations splenocytes were cultured as described above in the presence of 5 μg/ml of CPA_160-189_ or medium alone. At the last 4 h of incubation, cells were exposed to brefeldin A, washed in FACS buffer and stained with FITC-conjugated anti-CD4 or anti-CD8 mAbs (1:100 dilution; BD Biosciences) for 30 min. Then, cells were treated with permeabilization buffer and stained for 30 min on ice with PE-conjugated anti-IFN-γ mAb (1:100 dilution; BD Biosciences).

### Determination of CPA_160-189_-specific antibodies

Sera were collected from mice of all experimental groups 2 weeks after each vaccination (Table 1) and production of IgG, IgG1 and IgG2a antibodies against CPA_160-189_ was determined by ELISA. In brief, 96-well microtiter plates were coated with 5 μg/ml of CPA_160-189_ and incubated overnight at 4°C. After 3 washes with washing buffer (PBS with 0.05% Tween-20), plates were coated with PBS– 2% (w/v) BSA for 2 h. Then, sera samples were added at a dilution of 1:100 and incubated for 90 min. After that, biotinylated anti-mouse IgG1 (1 μg/ml) and IgG2a (250 ng/ml) (both obtained from AbD Serotec, Oxford, UK), or HRP-conjugated anti-mouse IgG (1:5000) (Thermo Scientific) were added for 1 h at 37°C. When biotinylated Abs were used, streptavidin-HRP was added (1:5000) and incubated for another 1 h at 37°C. The enzyme-labeled complexes were detected by reaction with TMB substrate and the reaction was stopped by adding 2 M sulfuric acid. The absorbance was measured at 450 nm using an ELISA microplate spectrophotometer (MRX).

### Statistical analysis

All results are expressed as mean±standard deviation (SD). GraphPad Prism version 5.0 software (San Diego, CA, USA) was used for statistical analysis. One-way ANOVA with multiple-comparisons, Tukey-Kramer post test or two-way ANOVA with Bonferroni post test were performed, when required, in order to assess statistical differences among experimental groups. A value of *p*<0.05 was considered significant for all analyses.

## Results

### CPA_160-189_ peptide is highly immunoreactive in the serum of naturally infected dogs with *L*. *infantum*

**Fig 1:**
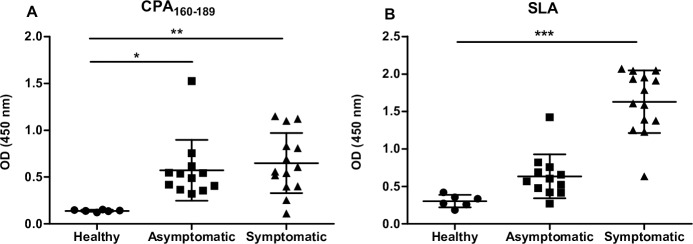
Evaluation of CPA_160-189_ immunogenicity in naturally infected dogs. Detection of (A) CPA_160-189_- and (B) SLA-specific IgG antibodies in sera samples from asymptomatic (n = 12) and symptomatic (n = 14) *L*. *infantum*-infected and non-infected healthy (n = 6) dogs. Lines indicate the mean±SD.

CPA is a highly immunoreactive molecule that is recognized by the sera obtained from either recovered or active cases of CL and VL, as well as by sera from asymptomatic or symptomatic dogs naturally infected with *L*. *infantum*. In order to determine whether CPA_160-189_ contains epitopes that are recognized by antibodies present in the serum of naturally infected dogs, an ELISA against CPA_160-189_ was performed. SLA of *L*. *infantum* was used as an internal control and a comparative antigen to CPA_160-189_. According to the results obtained, CPΑ_160–189_ peptide was recognized by highly reactive IgG antibodies present in both asymptomatic (OD values: 0.572±0.324, *p*< 0.05) and symptomatic (OD values: 0.674±0.322, *p*< 0.01) dog sera with a cut-off value of 0.173 (Fig 1), indicating the presence of antigenic epitopes of the synthetic peptide. As expected, healthy dogs recognized neither SLA nor CPA_160-189_ (Fig 1).

### Characterization of the synthesized PLGA NPs

**Fig 2:**
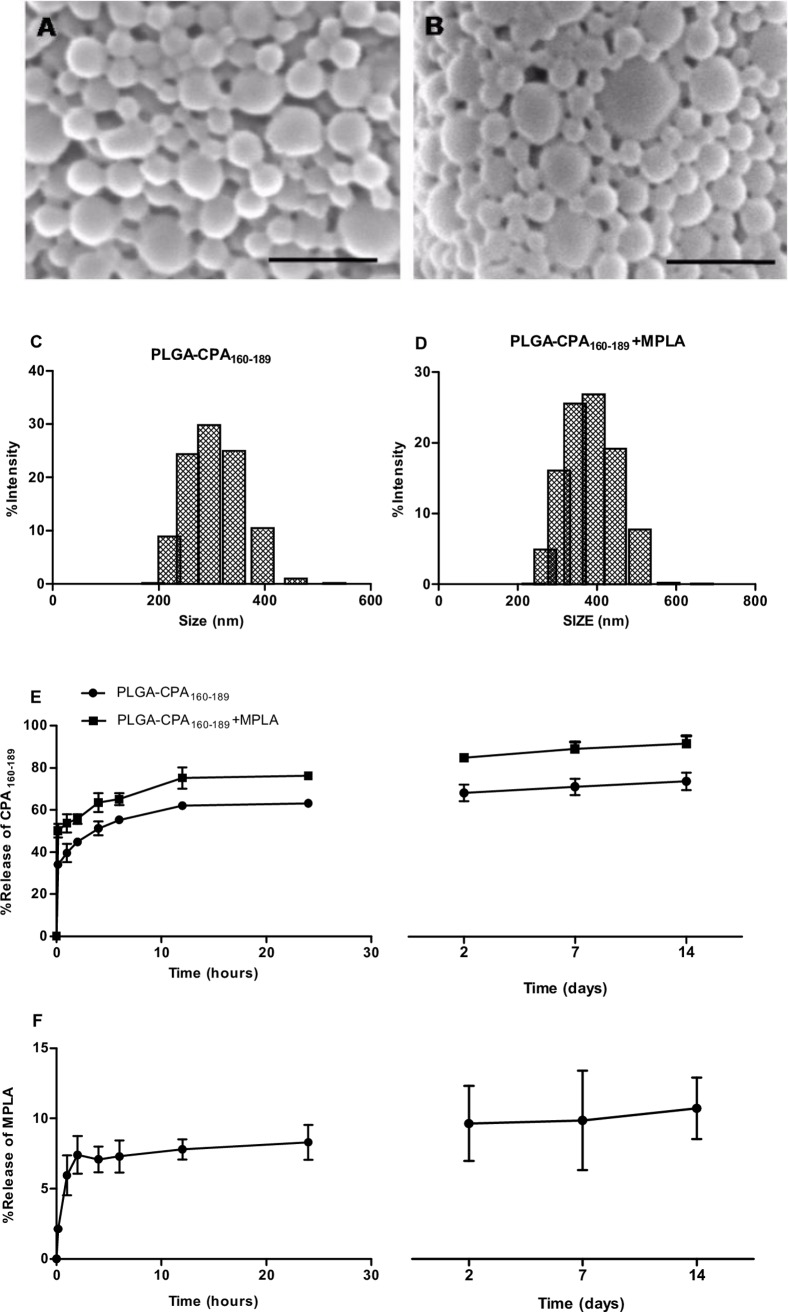
Characterization of the synthesized PLGA NPs. (A) PLGA-CPA_160-189_ NPs and (B) PLGA-CPA_160-189_+MPLA NPs were analyzed for their morphology by scanning electron microscopy analysis (SEM). Indicative SEM photomicrographs with scale bar at 1 μm. Size distribution of (C) PLGA-CPA_160-189_ NPs and (D) PLGA-CPA_160-189_+MPLA NPs. (E) CPA_160-189_ peptide and (F) MPLA *in vitro* release profile of PLGA-CPA_160-189_ NPs and PLGA-CPA_160-189_+MPLA NPs in PBS at 37°C. Data represent the mean±SD from three independent experiments.

Peptide-based vaccines may benefit from particulate delivery systems and the presence of the right adjuvant. For that reason, CPA_160-189_ was chosen to be co-encapsulated with MPLA into PLGA NPs serving as antigen vehicle. According to scanning electron microscopy, the synthesized PLGA NPs were characterized by a well-defined spherical shape (Fig 2A and 2B). The average diameter of different type of synthesized PLGA NPs was in the range of 309.9±15.4 to 316.6±6.6 nm as determined by photon correlation spectroscopy (% intensity) (Fig 2C and 2D) with a z potential varying from -18.2±7.7 to -19.1±5.7 mV (Table 2), indicative of the presence of free -COOH groups on the NP surface. The mean encapsulation efficiency (EE) of CPΑ_160–189_ ranged from 77.88±0.47 to 83.8±5.6%, and the antigen loading was 17±0.7 μg and 18.2±2.1 μg of CPΑ_160–189_ per mg of PLGA-CPA_160-189_ and PLGA-CPA_160-189_+MPLA NPs, respectively (Table 2). MPLA encapsulation in PLGA-CPA_160-189_+MPLA NPs was 80.1±8.2% and in PLGA-MPLA NPs was 54.5±3.1% corresponding to MPLA loading of 8.7±2.7 μg and 6.1±0.3 μg per mg of PLGA NPs, respectively (Table 2). *In vitro* release experiments at pH 7.4 revealed an initial burst release of CPA_160-189_ in both NPs formulations in the first 1 hour of the study reaching 39.5% and 53.7% in PLGA-CPA_160-189_ NPs and PLGA-CPA_160-189_+MPLA NPs, respectively, followed by a gradual increase until 24 hours of incubation (PLGA-CPA_160-189_ NPs: 63.1±1.5% and PLGA-CPA_160-189_+MPLA NPs: 76.2±0.6%). After that time point, antigen release profile was characterized by a plateau. Finally, after 2 weeks the cumulative percent of released CPA_160-189_ reached 69.6±3.9% and 86.56±3.4% in PLGA-CPA_160-189_ NPs and PLGA-CPA_160-189_+MPLA NPs, respectively (Fig 2E). In contrast, the percent of MPLA released in the same conditions reached only 8% after 24 h of incubation and remained stable until the end of the study (9.9±2%; Fig 2F).

**Table 2:** Properties of synthesized PLGA NPs.^a^.

NPs	Average size (nm)	PDI^b^	z potential (mV)	Ag loading (μg/mg)	Ag EE^c^ (%)	MPLA loading (μg/mg)	MPLA EE^c^ (%)
PLGA	312.8±3.8	0.06±0.03	-19.1±5.7	-	-	-	-
PLGA-MPLA	309.8±19.8	0.14±0.03	-18.8±2.1	-	-	6.1±0.3	54.5±3.1
PLGA-CPA_160-189_	309.9±15.4	0.08±0.02	-18.2±7.7	17±0.71	77.9±0.5	-	-
PLGA-CPA_160-189_+MPLA	316.6±6.6	0.15±0.01	-18.3±5.9	18.2±2.1	83.8±5.6	8.7±2.7	80.1±8.2

^a^Results are presented as mean±SD (n = 3), where appropriate.

^b^PDI: Polydispersity index.

^c^EE: Encapsulation efficiency.

### The most potent signatures of DCs maturation were obtained with the PLGA-CPA_160-189_+MPLA NPs vaccine

**Fig 3:**
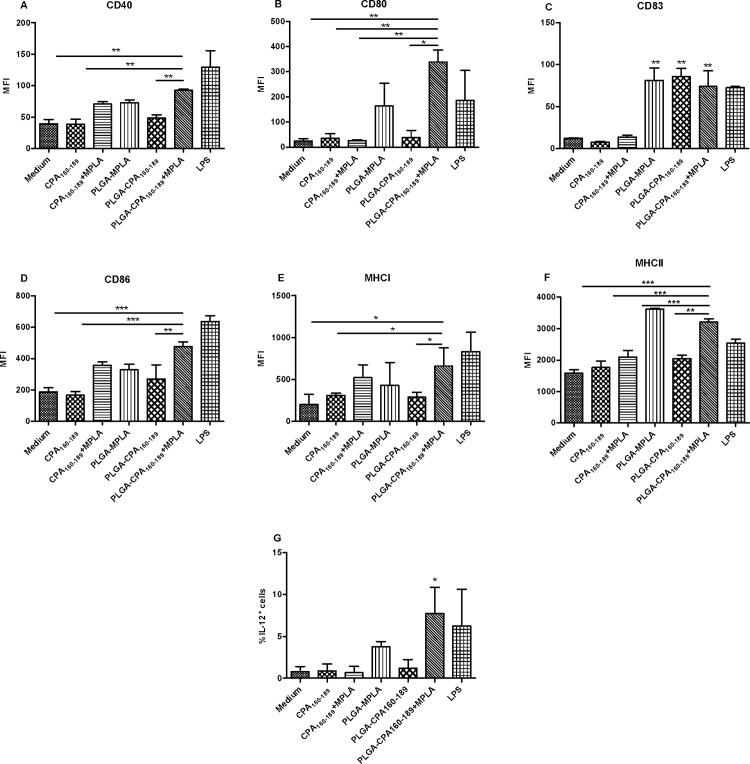
Co-encapsulation of CPA_160-189_ and MPLA in PLGA NPs induced activation of DCs. (A-F) Diagrams showing expression (MFI values) of CD40, CD80, CD83, CD86, MHCI and MHCII molecules on DCs and (G) (%) IL-12-producing DCs after pulsing with PLGA NPs. Results are expressed as mean±SD from three independent experiments. *p<0.05, **p<0.01, ***p<0.001 were assessed by one-way ANOVA and Tukey’s multiple comparison tests.

DCs play a central role in linking innate and adaptive immunity by successfully presenting antigens to T cells through MHC class I and/or II molecules. Moreover, MPLA, a synthetic analog of LPS, has the potential to activate DCs without the toxic effects of LPS, leading to activation of CD4^+^ Th1 and CD8^+^ T cell populations. In order to evaluate whether the uptake of the synthesized PLGA NPs could induce maturation of DCs, bone marrow-derived DCs were exposed to different PLGA NPs and the surface expression of CD40, CD86, CD80 and CD83 co-stimulatory and MHCI and MHCII molecules was determined by flow cytometry. For that reason, preliminary experiments were conducted in order to determine the optimum dose of PLGA NPs for efficient maturation of DCs assessed by CD40 and CD86 molecules expression. According to the results, PLGA-CPA_160-189_ NPs could not induce CD40 and CD86 co-stimulatory molecules expression, as assessed by low levels of MFI compared to DCs treated with medium alone (S1 Fig). In contrast, co-encapsulation of MPLA led to a dose-dependent upregulation of both maturation markers with the optimum dose of PLGA NPs ranging between 1 and 2 μg of CPA_160-189_ and 0.5 and 1 μg of MPLA, whereas at higher doses an advert effect on DCs maturation was observed (S1 Fig). For the purposes of the experiment it was selected the dose of PLGA NPs having co-encapsulated 2 μg of CPA_160-189_ and 1 μg of MPLA. As depicted in Fig 3, pulsing with PLGA-CPA_160-189_+MPLA NPs conferred significant increase not only in CD40 (92.5±1.8 vs 39.5±6.6, *p*<0.01) and CD86 (477.5±29.0 vs 188.1±27.1, *p*<0.001) co-stimulatory molecules expression but also in CD80 (339.5±47.38 vs 24.4±9.5, *p*<0.01), CD83 (74.1±18.53 vs 12.4±0.3, *p*<0.01), MHCI (660.5±218.5 vs 201.0±120.2, *p*<0.05) and MHCII molecules (3212.0±99.4 vs 1581.0±109.5, *p*<0.001) expression, as expressed by MFI values, compared to medium control (Fig 3A–3F). Interestingly, regarding CD83 expression both PLGA-MPLA NPs and PLGA-CPA_160-189_ NPs induced similar levels of increase compared to PLGA-CPA_160-189_+MPLA NPs in contrast to what happened to the other markers (PLGA-MPLA NPs: 81.2±14.99 vs PLGA-CPA_160-189_+MPLA NPs: 74.1±18.53; PLGA-CPA_160-189_ NPs: 85.9±9.76 vs PLGA-CPA_160-189_+MPLA NPs: 74.1±18.53) (Fig 3C). Also, it must be noted that the detected expression levels of all the above molecules, except CD86, were comparable to those detected in cells cultured in the presence of the positive control, LPS (CD40: 92.5±1.8 vs 129.0±26.87; CD80: 339.5±47.38 vs 186.5±118.1; CD83: 72.7±1.84 vs 74.1±18.53; CD86: 477.5±29.0 vs 635.5±63.0, *p*<0.05; MHCI: 660.5±218.5 vs 830.0±234.8; MHCII: 3212.0 ±99.4 vs 2542.0±242.0) (Fig 3A–3F). In contrast, the soluble mixture of peptide-adjuvant induced a minimal, not statistically significant increase in most of the maturation markers, except from CD80 and CD83, which was lower compared to that detected when DCs were pulsed with PLGA-CPA_160-168_+MPLA NPs (CD40: 70.8±5.2 vs 92.5±1.8, *p*<0.01; CD80: 26.1±3.5 vs 339.5±47.38, *p*<0.01; CD83: 13.80±2.12 vs 74.1±18.53, *p*>0.05; CD86: 358.5±21.9 vs 477.5±29.0, *p*>0.05; MHCI: 522.0±154.1 vs 660.5±218.5, *p*>0.05; MHCII: 2098±214 vs 3212±99.4, *p*<0.001) (Fig 3A–3F). Furthermore, pulsing with these PLGA NPs having co-encapsulated CPA_160-189_ and MPLA resulted to a significant increase of IL-12-producing DCs (7.7±3.1% vs 0.8±0.6%, *p*<0.05) compared to control (Fig 3G).

**Fig 4:**
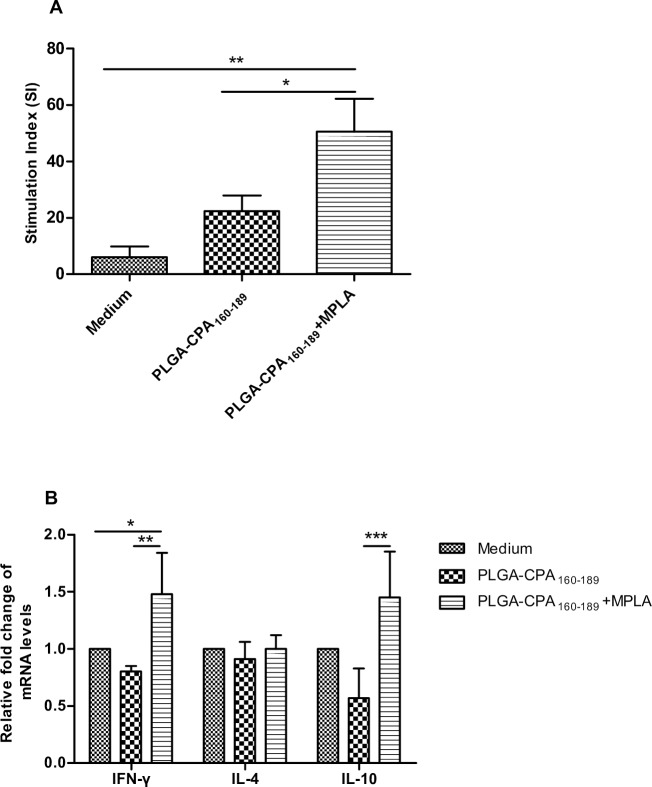
Antigen-presentation efficacy of PLGA NPs-pulsed DCs. (A) Antigen-presentation efficacy of PLGA NPs-pulsed DCs to naive splenocytes. Splenocytes treated with medium alone served as negative control. The results are the mean stimulation index (S.I.)±SD from three independent experiments. (B) IFN-γ, IL-4 and IL-10 mRNA expression in DCs-primed splenocytes by qRT-PCR. Results are presented as means±SD of three independent experiments. *p<0.05, **p<0.01, ***p<0.001 were assessed by one-way ANOVA and Tukey’s multiple comparison tests.

Subsequently, the capacity of these DCs to prime naive T cells by presenting CPA_160-189_ peptide was evaluated. To this end, DCs pulsed with the synthesized PLGA NPs were co-cultured with naive splenocytes of the same origin and their proliferation was assessed by ^3^[H]-thymidine incorporation. According to the results, DCs pulsed with PLGA-CPA_160-189_ NPs were capable of presenting the antigen since a 3.6-fold enhanced splenocyte proliferation was detected in comparison to splenocytes primed by DCs cultured in medium alone (SI: 22.4±5.5 vs 6.1±3.7, *p*>0.05) (Fig 4A). However, co-encapsulation of CPA_160-189_ with MPLA into PLGA NPs enhanced by 2-fold spleen cell proliferation compared to PLGA-CPA_160-189_ NPs (PLGA-CPA_160-189_+MPLA: 50.5±11.7 vs PLGA-CPA_160-189_: 22.4±5.5, *p*<0.05) (Fig 4A). In order to unveil whether DCs stimulated with the above PLGA NPs induced the differentiation and proliferation of peptide-specific Th1 or Th2 cell populations, analysis of mRNA expression for IFN-γ, IL-4 and IL-10 cytokines that are indicative of the respective T cell populations, was conducted. According to the results, priming of naive splenocytes with DCs that have been pulsed with the PLGA-CPA_160-89_+MPLA NPs stimulated significant upregulation of both IFN-γ and IL-10 transcripts by 1.5-fold (*p*<0.05) over those spleen cell cultures treated with immature DCs, whereas splenocytes that proliferated in the presence of PLGA-CPA_160-189_ NPs-pulsed DCs did not alter their IFN-γ and IL-10 expression compared to the control group (Fig 4B). On the contrary, IL-4 transcripts were not affected compared to the control group with both treatments (Fig 4B).

Taking together, these data indicated the requirement for the peptide and the adjuvant to be present in the same nanoparticulate formulation, since co-encapsulation of CPA_160-189_ and MPLA in PLGA NPs led to differentiation of IL-12-producing DCs and *in vitro* priming of CPA_160-189_-specific T cell populations.

### BALB/c mice after vaccination with PLGA NPs did not show any signs of toxicity and inflammation

Before processing to assessment of the synthesized PLGA NPs immunogenicity *in vivo*, their biocompatibility was investigated. All mice exhibited normal behavior throughout the study period. Moreover, induction of acute inflammation was not detected after the administration of all PLGA NPs, since IL-1β, IL-6, TNFα and MCP-1 production levels in the sera of vaccinated mice were detected at similar levels to the negative control group (Table 3) and were significantly lower to the positive control group injected with LPS.

**Table 3:** Detection of induction of acute inflammation after PLGA NPs injection.

Group	IL-1β (pg/ml)	IL-6 (pg/ml)	TNFα (pg/ml)	MCP-1 (pg/ml)
PLGA-CPA_160-189_	20.5±9.5	22±6.7	n.d.	112.5±9
PLGA-CPA_160-189_+MPLA	19.1±12.3	25.7±6.2	n.d.	98.3±28.4
PLGA-MPLA	32.6±19.8	140.4±61.7	n.d.	472.6±220.6
PBS (negative control)	29.8±8.1	13.6±4	n.d.	31.2±7
LPS (positive control)	40.3±8.9	22,808.5±733***	140.9±14.4***	15,557.1±1,700.5***

n.d.: Not detectable (under the detection limit of the assay).

***Significant different (*p*<0.001) as assessed by one-way ANOVA and Tukey’s multiple comparison tests.

### PLGA-CPA_160-189_+MPLA NPs-vaccinated BALB/c mice displayed CPA_160-189_-specific type 1 humoral and cellular immune responses

**Fig 5:**
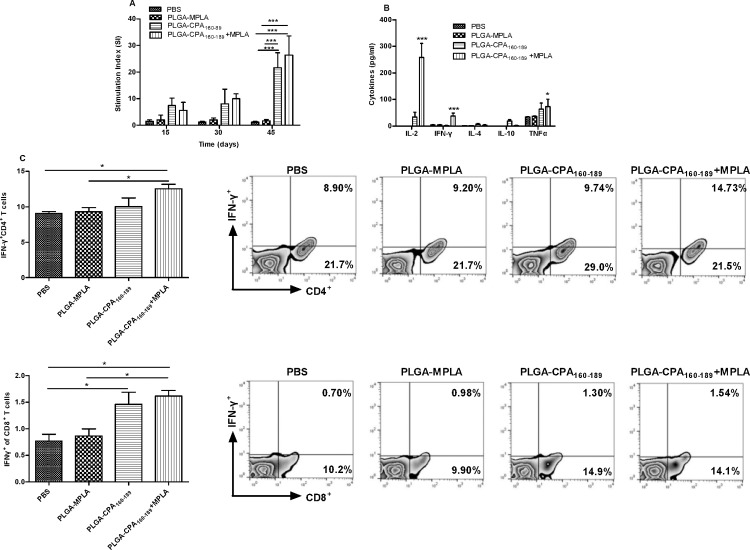
Determination of CPA_160-189_-specific cellular responses in PLGA NPs-vaccinated mice. (A) CPA_160-189_-specific proliferation of spleen cells obtained from mice vaccinated at different time points. Control group was vaccinated with PBS. Results are expressed as mean stimulation index (S.I.)±SD from three independent experiments. (B) CPA_160-189_-specific IL-2, IFN-γ, IL-4, IL-10 and TNFα production from spleen cells isolated two weeks after the end of vaccinations from vaccinated and non-vaccinated mice. (C) Diagram showing detection of CPA_160-189_-specific IFN-γ production by CD4^+^ and CD8^+^ T cells by flow cytometry analysis with representative dot plots, two weeks after the end of vaccinations. Results are presented as means±SD of five individual mice per group, representative of two independent experiments with similar results. *p<0.05, **p<0.01, ***p<0.001 for proliferation and cytokine assays were assessed by two-way ANOVA and Bonferroni’s multiple comparison tests, whereas flow cytometry results were assessed by one-way ANOVA and Tukey’s multiple comparison tests.

The efficiency of the synthesized PLGA NPs to induce CPA_160-189_-specific immune responses *in vivo* was analyzed. For this purpose, naive *Leishmania*-susceptible BALB/c mice were subcutaneously injected with the indicated doses of PLGA NPs and boosted twice in two weeks intervals (Table 1). According to the results, a single vaccination with PLGA NPs carrying CPA_160-189_ having co-encapsulated or not MPLA, elicited comparable levels of CPA_160-189_-specific splenocyte proliferation in contrast to PLGA-MPLA NPs-vaccinated mice and PBS control group which remained negative (PLGA-CPA_160-189_ NPs: 7.4±2.8; PLGA-CPA_160-189_+MPLA NPs: 5.6 ±3.0) (Fig 5A). Two booster doses, however, induced further increase in spleen cell proliferation with S.I. values against CPA_160-189_ reaching 21.6±5.7 (*p*<0.001) for splenocytes obtained from PLGA-CPA_160-189_ NPs-vaccinated mice and 26±7.2 (*p*<0.001) for splenocytes from PLGA-CPA_160-189_+MPLA NPs-vaccinated mice (Fig 5A). Moreover, the cytokine levels secreted by the splenocytes isolated from mice two weeks after the second boost in response to CPA_160-189_ stimulation were measured (Fig 5B). According to results, splenocytes obtained from mice vaccinated with PLGA-CPA_160-189_+MPLA NPs showed significantly enhanced production of IL-2 (258.59±52.13 pg/ml vs n.d., *p*<0.001) and the pro-inflammatory cytokines IFN-γ (37.67±11.23 pg/ml vs 4.42±1.8 pg/ml, *p*<0.001) and TNFα (73.22±28.08 pg/ml vs 34.1±1, *p*<0.05) in comparison to PBS control group (Fig 5B). This result indicated the differentiation of CPA_160-189_-specific effector T cells. On the contrary, vaccination with PLGA-CPA_160-189_ NPs induced substantial levels of IL-2 (33.87±18.2 pg/ml, *p*<0.05), TNFα (63.72±23.48 pg/ml, *p*<0.05) and the anti-inflammatory cytokine IL-10 (18.94±5.2 pg/ml) and not IFN-γ (Fig 5B). Regarding IL-4, both vaccinated groups produced low amounts after CPA_160-189_ stimulation as compared to PBS control group (PLGA-CPA_160-189_ NPs: 6.99±3.2 pg/ml vs 1.85±0.05, *p*<0.05 and PLGA-CPA_160-189_+MPLA NPs: 3.8±1.96 vs 1.85±0.05, *p*<0.05) (Fig 5B). Detection of IFN-γ-producing T cell populations showed that vaccination with PLGA-CPA_160-189_+MPLA NPs induced the differentiation of CPA_160-189_-specific IFN-γ-producing CD4^+^ T cells (12.55±0.64%, *p*<0.05) followed by PLGA-CPA_160-189_-vaccinated mice (10.05±1.2%) compared to PLGA-MPLA NPs-vaccinated and PBS control mice (PLGA-MPLA: 9.31±0.62% and PBS: 9.08±0.25%) (Fig 5C). Moreover, mice vaccinated with PLGA-CPA_160-189_ NPs and PLGA-CPA_160-189_+MPLA NPs induced about a 2-fold higher numbers of CPA_160-189_-specific IFN-γ-producing CD8^+^ T cells compared to the control group (PLGA-CPA_160-189_ NPs: 1.46±0.23% vs PBS: 0.77±0.13%, *p*<0.05 and PLGA-CPA_160-189_+MPLA NPs: 1.62±0.11% vs PBS: 0.77±0.13%, *p<0*.*05*) and PLGA-MPLA NPs-vaccinated mice (PLGA-CPA_160-189_ NPs: 1.46±0.23% vs PLGA-MPLA NPs: 0.87±0.13% and PLGA-CPA_160-189_+MPLA NPs: 1.62±0.11% vs PLGA-MPLA NPs: 0.87±0.13%, *p<0*.*05*) (Fig 5C).

**Fig 6:**
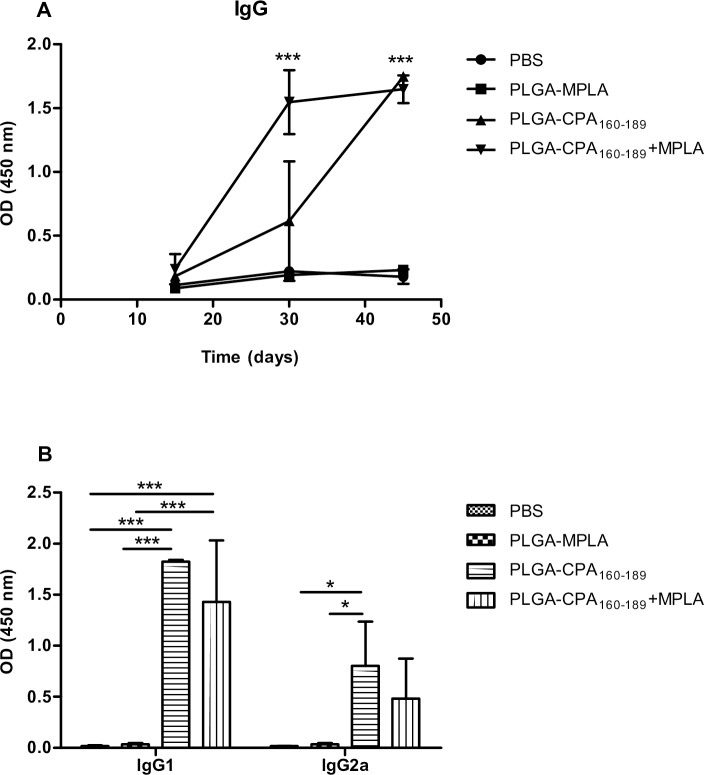
CPA_160-189_-specific humoral responses induction by PLGA NPs after vaccination. Detection of CPA_160-189_-specific (A) IgG, (B) IgG1 and IgG2a detection in mice sera vaccinated with PLGA NPs at the indicated time points. Results are presented as means±SD of five individual mice per group, representative of two independent experiments with similar results.*p<0.05, **p<0.01, ***p<0.001 for proliferation assay were assessed by two-way ANOVA and Bonferroni’s multiple comparison tests.

*In vitro* analysis of mice sera showed that PLGA-CPA_160-189_+MPLA NPs also generated secondary T-cell dependent sero-responses, since enhanced levels of peptide-specific IgG antibodies were detected after the second vaccination (1^st^ boost: 6-fold increase, *p*<0.001) over control groups, followed by PLGA-CPA_160-189_ NPs-vaccinated mice (1^st^ boost: 2.5-fold increase). However, at the end of vaccination both groups of vaccinated mice had comparable levels of CPA_160-189_-specific IgG antibodies (7-fold increase, *p*<0.001) (Fig 6A). Assessment of IgG1 and IgG2a antibodies showed that both isotypes were produced with a bias towards IgG1 (Fig 6B). Conclusively, the above results suggested that vaccination with PLGA-CPA_160-189_+MPLA NPs stimulated the activation and differentiation of CPA_160-189_-specific CD4^+^ Th1 and CD8^+^ T cell effector cells.

### Maintenance of CPA_160-189_-specific type 1 immune profile in the spleens obtained from mice 1 month post challenge with *L*. *infantum*

**Fig 7:**
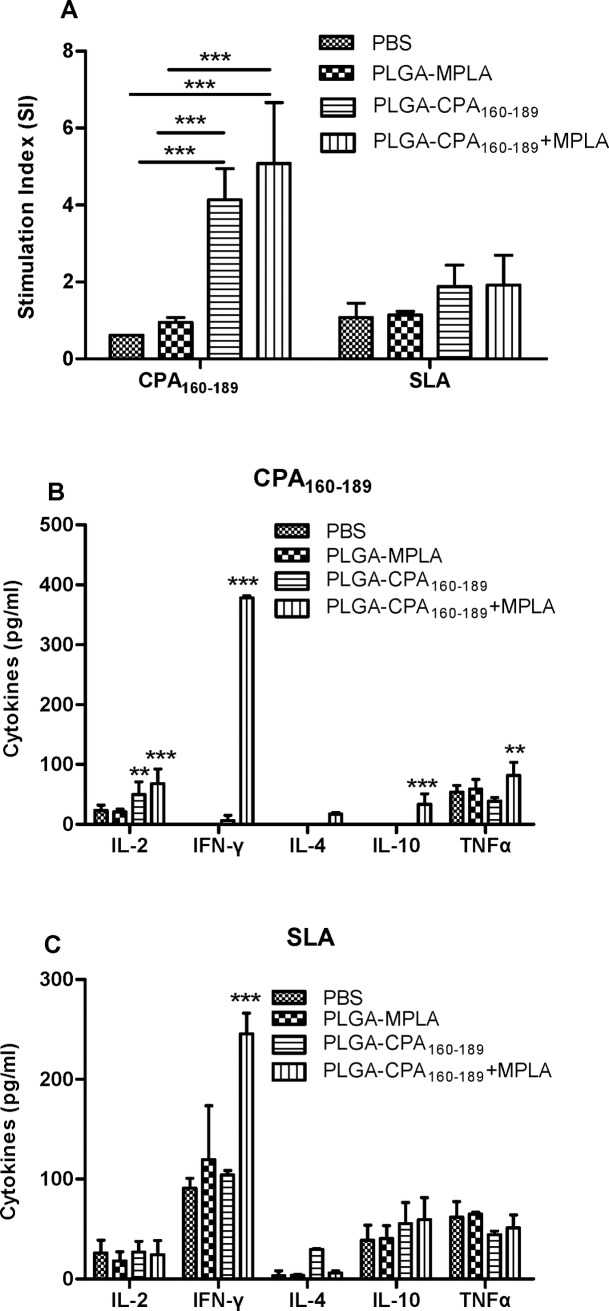
Cellular responses in PLGA NPs-vaccinated and *Leishmania*-infected mice. (A) CPA_160-189_- and parasite-specific proliferation and (B) CPA_160-189_- and (C) parasite-specific IL-2, IFN-γ, IL-4, IL-10 and TNFα production from spleen cells isolated 1 month post-challenge from vaccinated and non-vaccinated mice. Results are presented as means±SD of five individual mice per group, representative of two independent experiments with similar results. For cytokine production experiments background levels (medium alone) were subtracted. *p<0.05, **p<0.01, ***p<0.001 were assessed by two-way ANOVA and Bonferroni’s multiple comparison tests.

In order to evaluate whether the immune responses induced by vaccination were maintained during infection, the peptide-specific responses were assessed in both vaccinated and non-vaccinated mice infected with *L*. *infantum* one month post challenge. In conventional recall assays, splenocytes isolated from PLGA-CPA_160-189_ NPs and PLGA-CPA_160-189_+MPLA NPs-vaccinated mice showed comparable levels of significant peptide-specific proliferation (PLGA-CPA_160-189_ NPs: 4.1±0.8 vs PBS: 0.7±0.2, *p*<0.01; PLGA-CPA_160-189_+MPLA NPs: 5.1±1.6 vs PBS: 0.7±0.2, *p*<0.01). In contrast, spleen cells isolated from PLGA-MPLA NPs-vaccinated and non-vaccinated infected mice did not respond in the presence of CPA_160-189_ (Fig 7A). The observed immunosuppression in these mice groups extended also in parasite-specific immune responses, since only splenocytes obtained from both groups of PLGA-CPA_160-189_ NPs and PLGA-CPA_160-189_+MPLA NPs-vaccinated mice showed a 2-fold upregulation of lymphoproliferation in response to SLA compared to the non-vaccinated infected control group (PLGA-CPA_160-189_ NPs: 1.9±0.6 vs PBS: 1±0.4, PLGA-CPA_160-189_+MPLA NPs: 1.9±0.8 vs PBS: 1.0±0.4) (Fig 7A). Assessment of IL-2, IFN-γ, IL-4, IL-10 and TNFα production in the culture supernatant against CPA_160-189_ showed that the animals vaccinated with PLGA-CPA_160-189_+MPLA NPs produced enhanced levels of all cytokines after CPA_160-189_ treatment with an IFN-γ dominance (IL-2: 68.25±24.6 pg/ml, *p*<0.001; IFN-γ: 378.5±3.4 pg/ml, *p*<0.001; IL-4: 17.2±2.8 pg/ml, *p*<0.01; IL-10: 33.5±17.8 pg/ml, *p*<0.001; TNFα: 82.05±21.63 pg/ml, *p*<0.01) compared to the non-vaccinated infected control group which produced minimal amounts of IL-2 (23.11±9.29 pg/ml) and TNFα (53.88±11.47 pg/ml) cytokines in response to CPA_160-189_ stimulation. In contrast, PLGA-CPA_160-189_ NPs-vaccinated group was a low producer of IFN-γ (6.4±9.0 pg/ml) and produced mainly IL-2 (49.78±21.4 pg/ml, *p*<0.01) and TNFα (38.88±6.13 pg/ml) after stimulation with CPA_160-189_ (Fig 7B). Assessment of the parasite-specific immune responses showed that spleen cells from the mice that have been vaccinated with the PLGA-CPA_160-189_+MPLA NPs produced significantly higher amounts of IFN-γ compared to the PLGA-CPA_160-189_ NPs-vaccinated group (247.7±20.6 pg/ml vs 104.1±4.5 pg/ml, *p*<0.001) and the non-vaccinated infected control group (247.7±20.6 pg/ml vs 91.1±9.8 pg/ml, *p*<0.001), with minimal levels of IL-4 production (6.9±3.3 pg/ml vs 29.5±1.0 pg/ml, *p*<0.05) in response to SLA. In contrast, IL-2, IL-10 and TNFα levels were comparable to all groups tested (PLGA-CPA_160-189_+MPLA: IL-2: 24.4±14.3 pg/ml, IL-10: 59.7±21.9 pg/ml and TNFα: 51.3±12.9 pg/ml; PLGA-CPA_160-189_: IL-2: 27.2±10.3 pg/ml, IL-10: 55.6±21.1 pg/ml and TNFα: 44.5±3.3 pg/ml, PBS: IL-2: 26.1±12.7 pg/ml, IL-10: 38.9±14.9 pg/ml and TNFα: 61.8±15.7 pg/ml) (Fig 7C). Conclusively, PLGA-CPA_160-189_+MPLA NPs-vaccinated mice showed enhanced levels in CPA_160-189_- and parasite-specific IFN-γ production over IL-4 and IL-10 production resulting to high IFN-γ/IL-4 and IFN-γ/IL-10 ratios that indicated a predominance of Th1 immune responses. Moreover, the detection of CPA_160-189_-specific IL-2 and TNFα cytokines was indicative of the existence of effector T cell populations raised by vaccination with PLGA-CPA_160-189_+MPLA NPs. In contrast, splenocytes obtained from PLGA-CPA_160-189_ NPs- and PLGA-MPLA NPs-vaccinated and non-vaccinated infected mice groups were characterized by mixed parasite-specific Th1/Th2 immune responses and this was in accordance with the profile of VL characterized by such type of immune responses (Fig 7C).

### Vaccination with PLGA-CPA_160-189_+MPLA NPs induced partial protection against *L*. *infantum*

**Fig 8:**
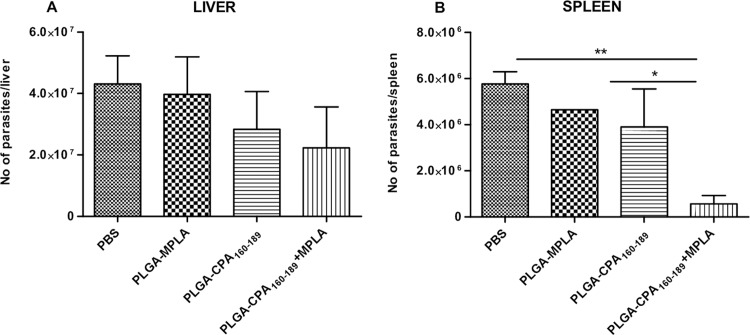
Evaluation of vaccine-mediated protection against *L*. *infantum* infection in BALB/c mice 1 month post intravenous challenge. Determination of parasite load in (A) the liver and (B) the spleen by limiting dilution assay in vaccinated and non-vaccinated mice 1 month post challenge with *L*. *infantum*. Results are presented as means±SD of five individual mice per group, representative of two independent experiments with similar results. *p<0.05, **p<0.01, ***p<0.001 were assessed by one-way ANOVA and Tukey’s multiple comparison tests.

**Fig 9:**
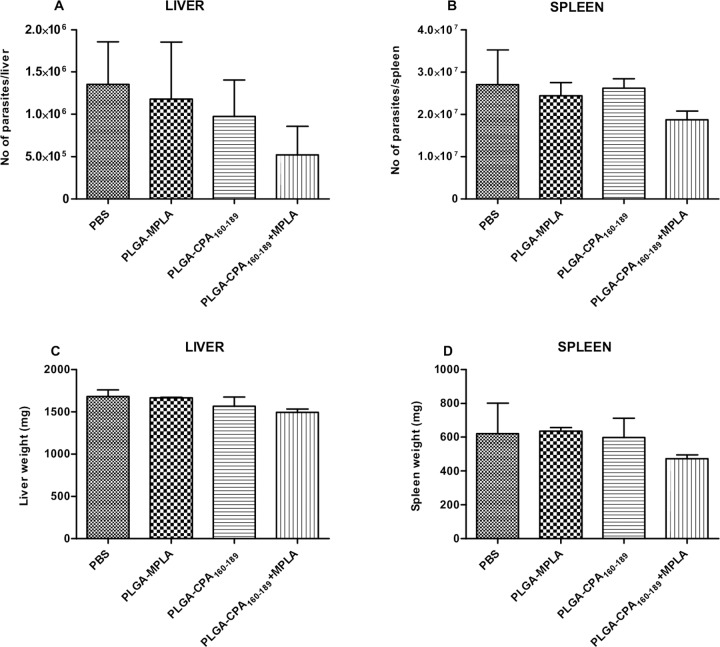
Evaluation of vaccine-mediated protection against *L*. *infantum* infection in BALB/c mice 4 months post intravenous challenge. Determination of (A-B) parasite load in the spleen and the liver by limiting dilution assay and (C-D) hepatosplenomegaly in vaccinated and non-vaccinated mice 4 months post challenge with *L*. *infantum*. Results are presented as means±SD of five individual mice per group, representative of two independent experiments with similar results. *p<0.05, **p<0.01, ***p<0.001 were assessed by one-way ANOVA and Tukey’s multiple comparison tests.

In order to elucidate whether the PLGA NPs-induced CPA_160-189_-specific T cells detected could confer protection against *Leishmania* parasites, the parasite burden was determined in liver and spleen 1 month later by limiting dilution assay. According to the results, vaccination reduced hepatic parasite burden by 34.2% and 48.2% in PLGA-CPA_160-189_ NPs- and in PLGA-CPA_160-189_+MPLA NPs-vaccinated mice, compared to the PBS control group, indicating that vaccinations could promote the self-curing response seen in BALB/c livers (Fig 8A). Moreover, evaluation of splenic parasite burden in mice vaccinated with PLGA-CPA_160-189_ NPs showed a 32.2% reduction when compared with non-vaccinated mice, which was further enhanced when mice were vaccinated with PLGA-CPA_160-189_+MPLA NPs resulting in the significant reduction in parasite load of 90.2% (*p*<0.01) (Fig 8B). Evaluation of parasite burden 4 months post challenge showed that although PLGA-CPA_160-189_+MPLA NPs-vaccinated mice preserved the reduced parasite burden in liver (61.5% reduction), an increase in the splenic parasite burden was detected (30.6% reduction) indicating a partial vaccine-induced protection (Fig 9A). These results were well correlated with spleen and liver weights as compared with the PBS control group (Fig 9B). In all cases, no effect of the vaccination with PLGA-MPLA NPs over infection was detected, confirming that the protection seen in liver and spleen through PLGA NPs was CPA_160-189_-specific.

## Discussion

Despite the fact that many attempts to design an effective vaccine against leishmaniasis have been made, when these experimental vaccines reached the clinical trials phase they failed to initiate strong T cell responses [3,4,5]. Difficulties in the design of such a vaccine for induction of T cell mediated immunity are originated not only from the high diversity and variability of the parasite, but also the HLA polymorphisms of the human population, thus affecting the specificities of T cell responses in different individuals. Bioinformatic analysis of protein sequences offers a solution towards this problem by designing synthetic peptides consisting of selected conserved epitopes among different parasite strains that are recognized by a number of different HLA molecules [33,34,35]. Accordingly, in the present study, the selected antigen to be used in the experimental vaccine was a 30-mer peptide, namely CPA_160-189_, designed by our research group to contain promiscuous overlapping human and murine MHC class I and II-restricted epitopes. CPA_160-189_ peptide’s immunogenicity was confirmed when administered in emulsion with Freund’s adjuvant in BALB/c mice, since it induced peptide-specific Th1 and CD8^+^ T cell responses [27].

As DCs play a central role in priming and controlling T cell mediated immune responses, efficient delivery of antigens to this cell population is a critical issue in the design of vaccine formulations for generation of effective cellular immune responses. In this context, nanoparticle delivery systems hold promise for achieving the appropriate type of immune responses. In the present study, the vaccine was designed in order to target effectively DCs, by using PLGA NPs with a relatively small size (~310 nm) to facilitate their uptake by APCs and free drainage to the lymphatic organs. According to previous studies, NPs with size smaller than 10 μm were more efficiently phagocytosed by APCs, including DCs, and trafficked towards the lymph nodes in a DC-dependent manner, which is an important aspect of vaccine delivery [36]. Indeed, in a recently published research of our group, we confirmed that these PLGA NPs were efficiently taken up by DCs *in vitro* and when were injected subcutaneously in mice they were phagocytosed by APCs in lymphoid organs, such as DCs [37].

Further, in the present study, it was shown that MPLA adjuvant encapsulation in PLGA NPs loaded with CPA_160-189_ was pivotal for triggering DCs functional maturation, as expressed by increased surface expression of co-stimulatory, MHC class I and II molecules and IL-12 production. It is known that MPLA-TLR4 interaction on the surface of DCs induces their functional activation characterized by maturation and expression of pro-inflammatory cytokines, such as IL-12, which are crucial for the activation of Th1 immune responses [38,39,40]. Moreover, the detection of enhanced CD83 expression levels on the surface of DCs triggered with PLGA-CPA_160-189_+MPLA NPs indicated the efficiency of these DCs to stimulate peptide-specific CD4^+^ and CD8^+^ T cells activation. Respectively, results of this study showed that *in vitro* priming of naive spleen cells with DCs pulsed with PLGA-CPA_160-189_+MPLA NPs induced the differentiation and activation of CPA_160-189_-specific T cells characterized by upregulated IFN-γ transcripts, further confirming the effectiveness of this delivery system for supporting polarized Th1 and/or CD8^+^ T cell immune responses through antigen presentation in context of MHC class II and/or MHC class I molecules. In a recent study, Maji et al showed the importance of adding the MPL adjuvant to liposomal rgp63 which led to a significant enhancement of the antigen presentation by DCs through TAP-dependent MHC class I pathway resulting in more efficient antigen-specific CD8^+^ T cell responses [13]. However, it must be noted that in our study, significant levels of IL-10 transcripts were also detected suggesting the activation of CPA_160-189_-specific T regulatory cell subpopulations.

Evaluation of PLGA NPs effectiveness to target DCs *in vivo* has been tested indirectly by assessing the development of CPA_160-189_-specific clones after mice vaccination. Despite the fact that mice received an extremely low dose (2 μg) of peptide, PLGA NPs effectively induced T and B cell CPA_160-189_-specific clonal expansion as indicated by recall assays and IgG detection, underlining the efficacy of PLGA NPs as peptide delivery system. Similar results have been observed in other studies using PLGA NPs for the development of experimental vaccines against cancer or various infections, such as malaria, and their efficacy to induce the desirable immune responses was attributed to the encapsulation of the MPLA adjuvant [41,42]. Interestingly, in contrast to *in vitro* results, vaccination with PLGA-CPA_160-189_ NPs led to similar levels of CPA_160-189_-specific T cell clonal expansion compared to that detected after vaccination with PLGA NPs loaded with CPA_160-189_ and MPLA. According to flow cytometry results, these proliferating splenocytes obtained from PLGA-CPA_160-189_+MPLA NPs-vaccinated mice contained both IFN-γ-producing CD4^+^ and CD8^+^ T cells. The above results confirmed and further extended our previous work, showing CPA_160-189_ peptide’s immunogenicity by eliciting a mixed Th1/Th2 response followed by almost equal CPA_160-189_-specific IgG2a and IgG1 production, in parallel with CD8^+^ T cells activation [27]. Possibly, CD8^+^ T cell activation detected in the present study was catalyzed by the presence of even low amounts of IL-4, as it was shown in the cytokine assay of the present study. The important role of IL-4 in the generation of CD8^+^ T cell memory against leishmaniasis has been shown in previous studies [43,44]. More specifically, the protective effect of different antigens, such as HASPB1 and histone H1, against VL was attributed to an IL-4-mediated activation of CD8^+^ T cells [45,46]. Moreover, splenocytes obtained from PLGA-CPA_160-189_+MPLA NPs-vaccinated mice produced also significant amounts of IL-2 and TNFα which along with IFN-γ suggest the existence of vaccine-induced effector T cell populations. These T cell populations are considered significant for vaccine efficacy to induce long-term protection against various pathogens, among them *Leishmania* [47,48].

In the area of nanoparticles vaccination, previous studies have shown that liposomal delivery of cysteine proteases CPA, CPB and CPC in combinations or alone, soluble leishmanial antigen and recombinant gp63 in the presence of MPLA adjuvant induced short and long-term immunity against VL due to the presence of both antigen-specific CD4^+^ and CD8^+^ T cell responses [49,50,51]. Also, it has been shown that when PLGA NPs were used as antigen vehicle in anti-tumor vaccines, a potent activation of antigen-specific CD8^+^ T cells was detected [52,53,54,55]. Since *Leishmania* antigen-specific CD4^+^ and CD8^+^ T cells are essential for immunity against leishmaniasis [56,57], the protective effect of these populations detected in the present study was assessed by challenging vaccinated mice with a highly virulent strain of *L*. *infantum* promastigotes. The immune response elicited by PLGA-CPA_160-189_+MPLA NPs vaccination conferred significant reduction of parasite burden in spleen and liver by 90% and 40%, respectively. This decrease was positively correlated with the enhanced proliferation of splenocytes in response to CPA_160-189_ and SLA stimulation in comparison to control infected groups.

Determination of cytokine profile showed high levels of IL-2, IFN-γ and TNFα production contrary to the minimal levels of IL-4 and IL-10 leading to enhanced IFN-γ/IL-4 and IFN-γ/IL-10 ratios. It is well documented that whereas IL-4 does not play a decisive role in visceral infection establishment, IL-10 production by splenic cells correlates well with disease progression and pathology in human and experimental disease [58,59,60]. IL-10 has been shown to block Th1 activation and consequently cytotoxic response by down-regulating IFN-γ levels. Further, it decreases the ability of macrophages to destroy parasite by deactivating them. On the other hand, protective immunity against VL is dependent on IL-12-driven Th1 immune response and IFN-γ synergizes with TNFα resulting in the induction of parasite killing by macrophages [61]. However, the simultaneous existence of IL-2, IFN-γ and TNFα producers was followed by a partial protection, since determination of parasite load at 4 months post challenge showed that mice had limited vaccine efficacy to restrain uncontrolled parasite expansion in spleen with only 30% reduction, whereas a decrease in hepatic parasite burden was observed (61%). These results, in contrast to previous findings [47,48], indicated that CPA_160-189_-induced cell immune responses were not capable for the maintenance of long-term protective immunity against the parasite.

According to studies exploring CPA’s potential as an effective vaccine against leishmaniasis, CPA conferred significant protection in the experimental models of CL and VL, as wells as in the experimental model of canine leishmaniasis when was administered with other immunogenic proteins [62,63,64,65]. However, the fact that a single peptide of CPA (CPA_160-189_) co-encapsulated with MPLA in PLGA NPs, without the presence of peptides extracted from other immunogenic parasitic molecules, proved to confer significant protection further supporting the appropriateness of the current strategy for the peptide design. Furthermore, the proposed vaccine could be improved by encapsulating more than one synthetic peptides obtained from different *Leishmania* proteins in order to achieve more intense T-cell responses, since previous studies support that vaccines that address a broad range of specificities are capable of inducing polyclonal effector T cells promoting protection [64]. In addition, in the light of recent findings, anti-leishmanial vaccine efficacy could be improved by including vector-derived molecules in combination with parasitic molecules [66].

Taken together, the data of the present study could provide the basis for the development of peptide-based nanovaccines against leishmaniasis, since it reveals that vaccination with nanovaccines that contain rationally designed multi-epitope peptides covering areas that interact both with MHC class I and II molecules in combination with the appropriate adjuvant and biocompatible delivery system could be a promising approach for the induction of desirable protective immune responses.

## Supporting Information

S1 Fig Dose–dependent PLGA NPs effect on DCs maturation. Analysis of (A) CD40 and (B) CD86 expression (MFI values) on DCs pulsed with PLGA NPs at different doses with flow cytometry. Results are expressed as mean±SD (n = 3) from three independent experiments. *p<0.05, **p<0.01, ***p<0.001 were assessed by one-way ANOVA and Tukey’s multiple comparison tests.
(TIF)

S2 Fig Effect of PLGA NPs-pulsing on DCs functional maturation. (A) Representative histograms and (B) dot plots showing CD40, CD80, CD86, MHCI and MHCII molecules expression and (%) of IL-12-producing DCs after treatment with PLGA NPs.
(TIF)
