# Supplementary figures and images for: Identification of BALB/c Immune Markers Correlated with a Partial Protection to Leishmania infantum after Vaccination with a Rationally Designed Multi-epitope Cysteine Protease A Peptide-Based Nanovaccine
Source: PLoS Negl Trop Dis. 2017 Jan 23;11(1):e0005311. doi: 10.1371/journal.pntd.0005311 (PMC5295723; doi:10.1371/journal.pntd.0005311)

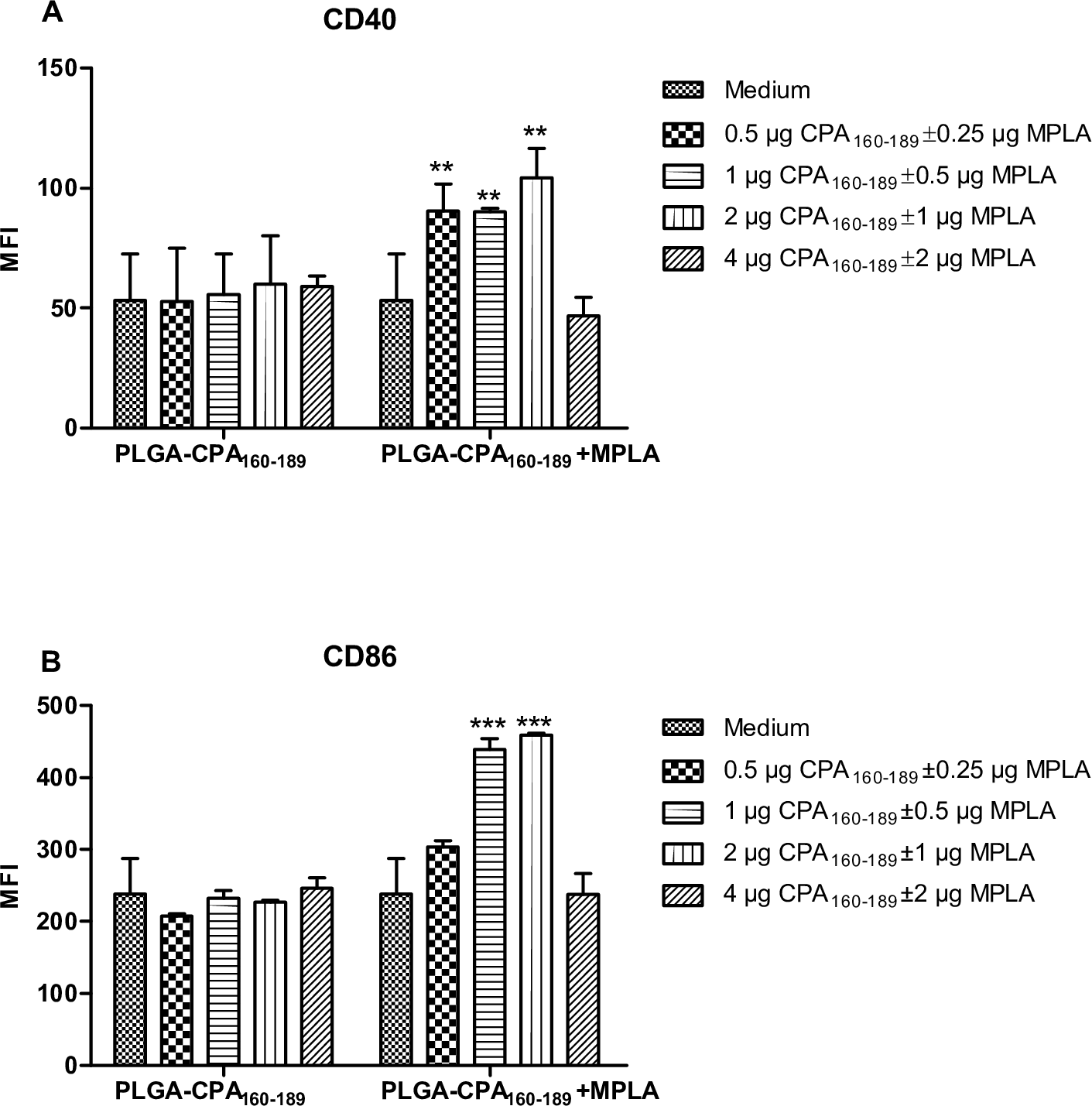

Supplement: S1 Fig — Analysis of (A) CD40 and (B) CD86 expression (MFI values) on DCs pulsed with PLGA NPs at different doses with flow cytometry. Results are expressed as mean±SD (n = 3) from three independent experiments. *p<0.05, **p<0.01, ***p<0.001 were assessed by one-way ANOVA and Tukey’s multiple comparison tests. (TIF) [file pntd.0005311.s001.tif]

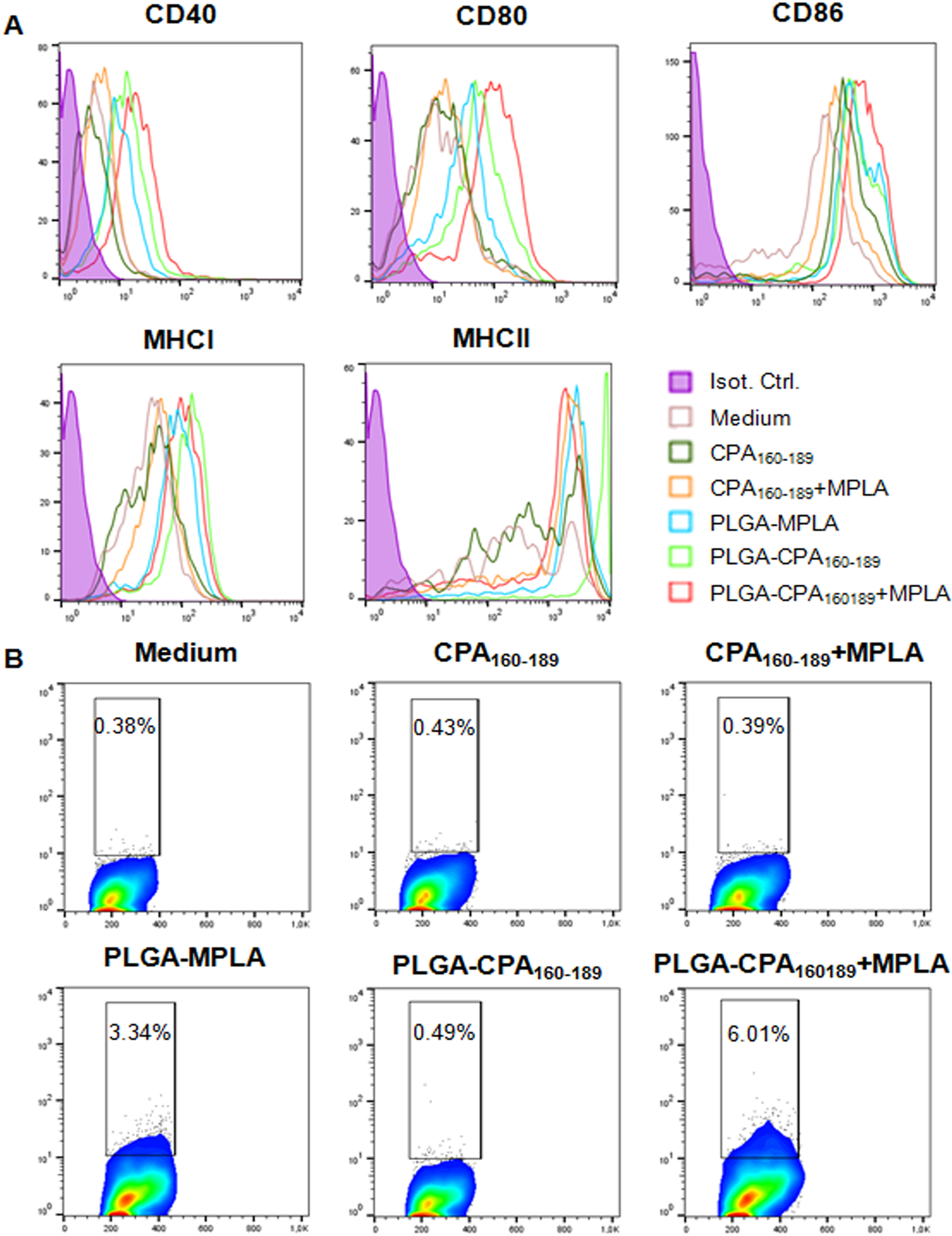

Supplement: S2 Fig — (A) Representative histograms and (B) dot plots showing CD40, CD80, CD86, MHCI and MHCII molecules expression and (%) of IL-12-producing DCs after treatment with PLGA NPs. (TIF) [file pntd.0005311.s002.tif]
